# Is myocardial bridge more frequently detected on radial access coronary angiography?

**DOI:** 10.1186/s12872-021-02382-y

**Published:** 2021-11-23

**Authors:** Oktay Şenöz, Zeynep Yapan Emren

**Affiliations:** Department of Cardiology, Bakırcay University Cigli Training and Research Hospital, 35550 Cigli, Izmir Turkey

## Abstract

**Background:**

Although the incidence of myocardial bridge (MB) has been defined in different femoral access conventional coronary angiography (FACCA) studies, the frequency of MB on radial access coronary angiography (RACA) is unknown. The aim of this study was to determine the difference in the incidence of MB between patients undergoing RACA and FACCA.

**Method:**

A total of 2500 consecutive patients who underwent RACA and a total of 1455 consecutive patients who underwent FACCA were retrospectively investigated to detect the presence of MB. The incidences of the groups were calculated separately and compared. The clinical and angiographic features of the patients with MB were analyzed.

**Results:**

MB was detected at an incidence of 10.2%, in 255/2500 patients who underwent RACA, and 1.8% in 27/1455 patients who underwent FACCA (*p* < 0.001). In both RACA and FACCA patients, the most involved coronary artery was the left anterior descending artery (LAD) (86.9% and 93.1%) and the mid-segment (84.9% and 88.9%) was the most affected section. Co-involvement of multiple coronary arteries by MB was 7.8% in patients who underwent RACA and 7.4% in patients who underwent FACCA. Coronary artery disease (CAD) was determined in 111 (35.7%) of the coronary arteries with MB, of which 81.9% were proximal to the MB. No significant CAD was detected in any of the vessels of 69.8% (178/255) of the patients who underwent RACA for different clinical indications.

**Conclusion:**

These data demonstrated that the incidence of myocardial bridge able to be detected on RACA was much higher than FACCA.

## Introduction

Myocardial bridge (MB) is an anatomic variation in which some of the epicardial coronary arterial segments run into the myocardium. MB is characterized by narrowing during the systole of the coronary artery by overlying muscle fibers [1, 2]. It usually occurs in the mid-segment of the left anterior descending coronary artery (LAD) [3, 4]. MB may affect other coronary arteries less frequently and occasionally, all of them [5]. Although MB is known to be usually benign, it may sometimes cause myocardial ischemia, arrhythmia, syncope, and sudden cardiac death [6, 7]. Coronary heart disease can be caused by MB both by direct compression in cardiac systole and by exacerbation of atherosclerosis progression in the vessel proximal to the MB [8, 9]. There is a great difference in the incidence of MB reported in angiographic series (0.5–2.5%) and in autopsy series (15–85%) [10]. It has been shown that the frequency and extent of MB may differ according to imaging techniques. In comparative studies of the same patient population, the frequency of MB was 6% with conventional coronary angiography, and 30% with computed tomography coronary angiography (CTCA) [11]. Those results confirmed that conventional coronary angiography is not sensitive enough to detect MB, especially of a mild type [12]. Radial access for coronary angiography has been shown to reduce major bleeding and ischemic events compared to femoral access [13]. Therefore, radial access has been the principal approach for coronary angiography in recent years. The aim of this study was to determine the difference in the incidence of MB between patients undergoing RACA and FACCA.

## Method

### Study population

A retrospective evaluation was made of the coronary angiographies of 2500 consecutive patients who underwent RACA and 1455 consecutive patients who underwent FACCA between January 2018 and February 2021. The incidence of MB in patients who underwent RACA and FACCA was calculated separately and their incidences were compared. Patients with a history of coronary artery bypass grafting (CABG) were excluded from the study. The clinical and angiographic features of patients with MB were analyzed.

Patients who underwent RACA or FACCA for the diagnosis of coronary artery disease were grouped according to clinical conditions as non-anginal symptoms, stable angina pectoris (SAP), unstable angina pectoris (USAP), non-ST-segment elevation myocardial infarction (NSTEMI), and ST-segment elevation myocardial infarction (STEMI). Cardiac single-photon emission computed tomography (SPECT) or treadmill exercise test was performed before coronary angiography in some patients who presented with stable angina pectoris and non-anginal symptoms. The SPECT was positive in 24 and the treadmill exercise test was positive in 60 of RACA patients. Patients who underwent elective coronary angiography due to stable angina pectoris and non-anginal symptoms fasted at least 12 h before and their medications were interrupted. However, this condition was not complied with in patients who underwent urgent or early angiography due to myocardial infarction.

### Radial access procedure

The right radial artery was cannulated with a 6-f radial sheath after local infiltration with 2% lidocaine. All patients received 2500 to 5000 units of unfractionated heparin according to weight and glomerular filtration rate, 100–200 μg (depending on blood pressure) of nitroglycerin, and 5 mg of diltiazem unless there was an absolute contraindication to diltiazem and anticoagulants. Coronary angiography was performed using the standard Judkins` technique via right radial access with a 5-f diagnostic catheter. Standard angiography images were obtained with a biplane cine-angiography system. Each angiogram was reviewed by the same two qualified cardiologists.

### Definitions and measurements

The diagnosis of SAP was based on the presence of chest pain that did not change its pattern during the preceding 2 months. USAP was defined as chest pain at rest, or marked progression of known angina within the last 2 weeks or recent and progressive onset of angina with evidence of ischemia on the ECG. NSTEMI was defined as an elevation of high sensitive troponin T level ≥ 14 ng/L, accompanied by either typical chest pain for > 30 min and/or electrocardiographic change. STEMI was defined as > 30 min of continuous typical chest pain and ST-segment elevation of 1 mm in at least 2 limb electrocardiographic leads or 2 mm in at least 2 contiguous precordial leads or the presence of a new left bundle branch block. For the diagnosis of hypertrophic obstructive cardiomyopathy, it was required that the ratio of septum thickness to the posterior wall was ≥ 1.3 and the left ventricle outflow tract gradient at rest and/or stimulation was ≥ 30 mmHg.

The presence of MB was defined as the narrowing of the coronary artery lumen in systole and expansion in diastole with no evidence of coronary vasospasm. The extent and severity of MB and its relationship with coronary artery disease (CAD) were examined on cine-angiograms. The quantification of systolic lumen compression and atherosclerotic stenosis in the coronary artery was performed using a digital caliper program to measure the lumen diameter reduction. With this digital program, vessel inner diameter was measured in diastole (expansion) and systole (contraction). Measurements were performed in the left anterior oblique position. The percentage of systolic lumen narrowing caused by MB was calculated with the following equation: (diastolic diameter − systolic diameter) × 100/diastolic diameter. To determine the severity of MB, patients were divided into 3 groups according to the degree of systolic lumen compression.

- Group—1 (mild): Systolic lumen narrowing ≤ 50%,
- Group—2 (moderate): Systolic lumen narrowing between 51 and 70%,
- Group—3 (severe): Systolic lumen narrowing ≥ 71%.

The patients were also separated into 3 groups according to the degree of luminal narrowing caused by atherosclerotic stenosis.

- Group—1 (mild): Luminal narrowing between 30 and 50%,
- Group—2 (moderate): Luminal narrowing between 51 and 70%,
- Group—3 (severe): Luminal narrowing ≥ 71%.

Severe CAD was defined as the presence of ≥ 71% coronary artery stenosis or the presence of a previously placed stent due to severe coronary artery stenosis. Significant CAD was defined as the presence of ≥ 51% coronary artery stenosis.

### Statistical analysis

Data obtained in the study were analyzed statistically using SPSS for Windows, vn.15.0 (SPSS Inc, Chicago, IL, USA). Conformity of the data to normal distribution was assessed using the Kolmogorov–Smirnov test. Continuous variables were reported as mean ± standard deviation (SD), minimum and maximum or median (interquartile range Q1–Q3) according to distribution normality, and categorical variables as number (n) and percentage (%). The groups were compared using independent Student’s T-test or Mann–Whitney U test for continuous variables based on normality distribution, and Chi-squared test or Fisher’s exact test for categorical variables. A value of *p* < 0.05 was accepted as statistically significant.

## Results

**Fig. 1:**
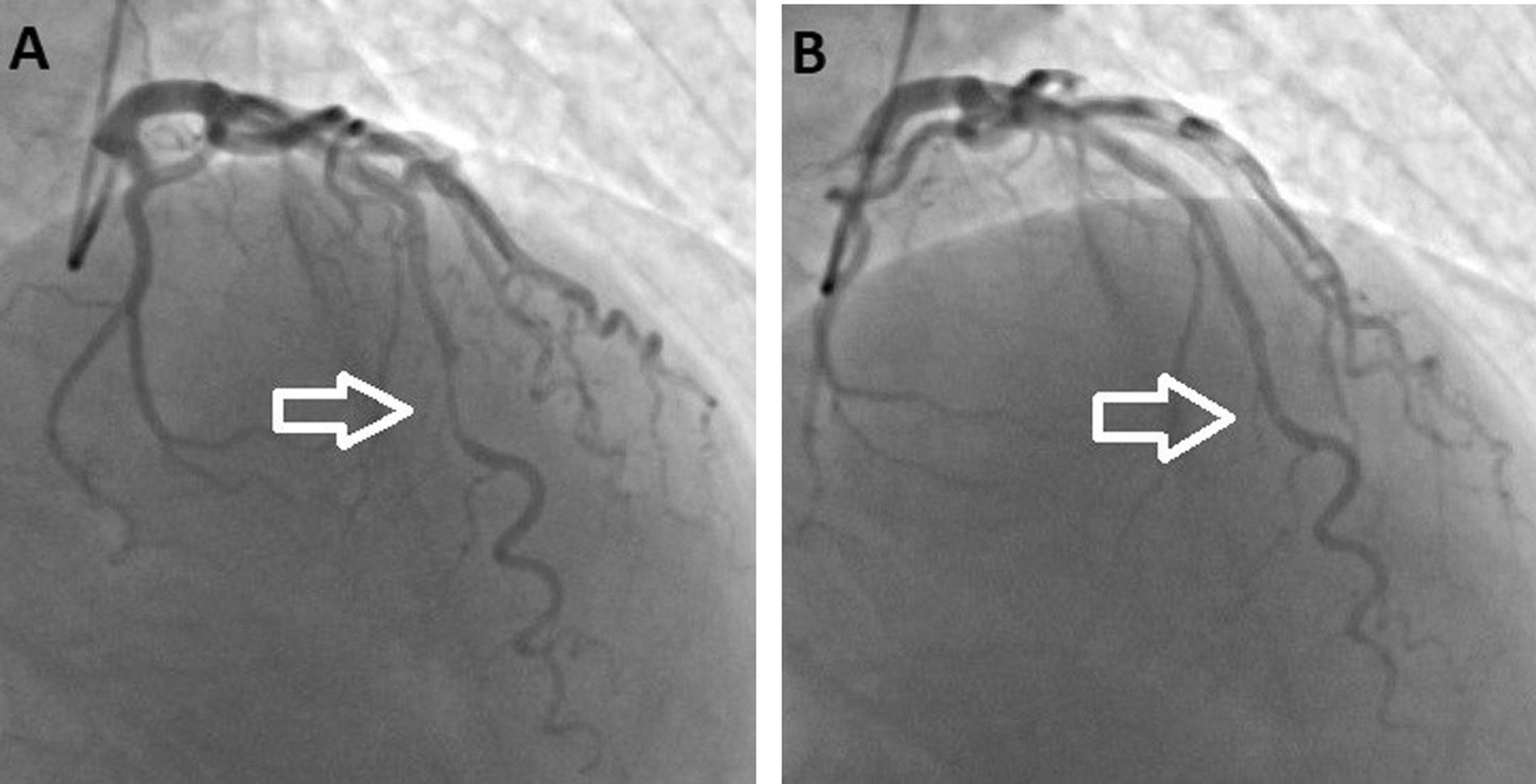
Severe degree MB in LAD, arrows show systolic compression (**A**) and diastolic expansion (**B**)

MB was detected in 255 of 2500 patients who underwent RACA and 27 of 1455 patients who underwent FACCA, giving a total incidence of 10.2% and 1.8% respectively. The incidence of MB was significantly higher in patients undergoing RACA than those undergoing FACCA (*p* < 0.001). The 282 patients comprised 215 (76.2%) males and 67 (23.8%) females with a mean age of 57.8 ± 11.1 years (range, 25–83 years; median, 59 years). Hypertension was determined in 154 (54.6%) patients. The echocardiographic findings showed left ventricular concentric hypertrophy (LVCH) in 93 patients, hypertrophic obstructive cardiomyopathy (HOCM) in 3, and aortic stenosis (AS) in 5. While the majority of RACAs were performed for some non-urgent clinical conditions (non-anginal symptoms, stable angina, and unstable angina), the majority of FACCAs were performed urgently for myocardial infarctions (*p* < 0.001).The demographic data and clinical features of the patients are presented in Table 1. While 235 (92.2%) patients who underwent RACA had single-vessel MB and 20 (7.8%) had more than one vessel MB (two vessels in 13 patients, three vessels in 7), 25 (92.6%) patients who underwent FACCA had single-vessel MB and 2 (7.4%) had two-vessel MB. There were 282 coronary arteries with MB in RACA patients, and 29 coronary arteries with MB in FACCA patients. In both RACA and FACCA patients, the most involved coronary artery was LAD (86.9%, 245/282 and 93.1%, 27/29) and the most affected section of LAD was the mid-segment (84.9%, 208/245 and 88.9%, 24/27) followed by distal (13.9%, 34/245 and 11.1%, 3/27) (Fig. 1). The median length of MBs was 20 (15–25.25) mm on RACA and 22 (18.5–24) mm on FACCA. Mild degree MBs were most common in both RACA and FACCA patients, 45.9% and 48% respectively. Angiographic characteristics of the patients according to the access site are presented in Table 2. In addition, detailed angiographic features of RACA patients according to MB grades are presented in Table 3. Of the MB-related coronary arteries, 35.7% (111/311) had CAD, of which 81.9% were proximal to the bridge, 9.1% were in the bridge segment, and 9.1% were distal to the bridge. Of these CADs, 81 (72.9%) were mild, 4 (3.6%) moderate, and 26 (23.4%) severe.

**Table 1:** Demographic and clinical characteristics of the patients

Variables	RACA patients (n = 255)	FACCA patients (n = 27)	*p* value
Age, years (mean ± SD)	57.5 ± 11.3	60.9 ± 11.2	0.129
Male gender, n (%)	196 (76.9)	19 (70.4)	0.451
Hypertension, n (%)	137 (53.7)	17 (65.4)	0.255
Diabetes mellitus, n (%)	63 (24.7)	10 (37)	0.168
Smoking, n (%)	104 (40.8)	11 (40.7)	0.984
Hypercholesterolemia, n (%)	127 (50.4)	17 (63)	0.214
Chronic renal failure, n (%)	20 (7.8)	3 (11.1)	0.555
CVD history, n (%)	5 (1.9)	2 (7.7)	0.075
HOCM, n (%)	2 (0.8)	1 (4.2)	0.241
LVCH, n (%)	86 (33.7)	7 (29.2)	0.605
Aortic stenosis, n (%)	4 (1.6)	1 (4.2)	0.370
LVDD, n (%)	188 (73.7)	18 (75)	0.983
LVEF, % (mean ± SD)	57.7 ± 6	56.4 ± 5.8	0.349
*Admission clinic, n (%)*			
Non-anginal symptoms	59 (23.1)	2 (7.4)	**< 0.001**
Stable angina	117 (45.9)	4 (14.8)	
Unstable angina	33 (12.9)	5 (18.5)	
Anterior MI	3 (1.2)	2 (7.4)	
Inferior MI	5 (1.9)	7 (25.9)	
NSTEMI	38 (14.9)	7 (25.9)	
*Arrhythmia, n (%)*			
Atrial	3 (1.2)	1 (3.7)	0.333
Ventricular	2 (0.8)	1 (3.7)	0.261

The p-value is statistically significant in bold

*SD* standard deviation, *n* number of patients, *CVD* cerebrovascular diseases, *FACCA* femoral access conventional coronary angiography, *HOCM* hypertrophic obstructive cardiomyopathy, *LVCH* left ventricular concentric hypertrophy, *LVDD* left ventricular diastolic dysfunction, *LVEF* left ventricular ejection fraction, *MI* myocardial infarction, *NSTEMI* non-ST segment elevation myocardial infarction, *RACA* radial access coronary angiography

**Table 2:** Distribution of MBs on coronary arteries and angiographic characteristics of the patients according to access cites

Variables	RACA patients (n = 255)	FACCA patients (n = 27)	*p* value
*Coronary arteries with MB*			
LAD, n (%)	225 (88.2)	25 (92.6)	0.497
CX, n (%)	8 (3.1)	0	0.350
RCA, n (%)	2 (0.8)	0	0.644
LAD and CX, n (%)	9 (3.5)	2 (7.4)	0.322
LAD and RCA, n (%)	4 (1.6)	0	0.512
LAD, CX, and RCA, n (%)	7 (2.7)	0	0.383
*Angiographic characteristics of the single-vessel MB patients*			
Length of MB, mm, (median (IQR Q1–Q3))	20 (15–25.25)	22 (18.5–24)	0.897
MB degree, n (%)			
Mild	108 (45.9)	12 (48)	0.866
Moderate	59 (25.1)	7 (28)	
Severe	68 (28.9)	6 (24)	
Affected segment of vessel from MB, n (%)			
Proximal	4 (1.7)	0	0.805
Mid	204 (86.8)	22 (88)	
Distal	27 (11.5)	3 (12)	

*n* number of patients, *CX* circumflex artery, *FACCA* femoral access conventional coronary angiography, *LAD* left anterior descending artery, *MB* myocardial bridge, *RACA* radial access coronary angiography, *RCA* right coronary artery, *Q* quartiles

**Table 3:** Angiographic features of single-vessel MB patients undergoing RACA according to MB grades

Variables	Group 1 (MB < 50%) (n: 108)	Group 2 (51% < MB < 70%) (n: 59)	Group 3 (MB ≥ 71%) (n: 68)	Total (n: 235)
LAD, n (%)	100 (92.6)	57 (96.6)	68 (100)	225 (95.7)
CX, n (%)	6 (5.6)	2 (3.4)	0	8 (3.4)
RCA, n (%)	2 (1.9)	0	0	2 (0.9)
Length of MB, mm (median (IQR Q1–Q3))	17 (13–22.75)	23 (19–27.75)	22 (17.25–32.75)	20 (15–25.25)
*Affected segment of vessel from MB, n (%)*				
Proximal	1 (0.9)	2 (3.4)	1 (1.5)	4 (1.7)
Mid	92 (85.2)	51 (86.4)	61 (89.7)	204 (86.8)
Distal	15 (13.9)	6 (10.2)	6 (8.8)	27 (11.5)

*n* number of patients, *CX* circumflex artery, *IQR* interquartile range, *LAD* left anterior descending artery, *MB* myocardial bridge, *RCA* right coronary artery, *Q* quartiles

No significant CAD was detected in any of the vessels of 59 (70.2%) patients who underwent RACA due to SPECT or treadmill exercise test positivity. Of these 59 patients, 28 had mild, 17 moderate, and 14 severe MB. In addition, no significant CAD was detected in any of the vessels of 69.8% (178/255) of all patients who underwent RACA for different clinical indications. The clinical presentations of these patients according to their MB grades are presented in Table 4.

**Table 4:** Clinical presentations of patients undergoing RACA without significant coronary artery disease according to MB grades

Variables	Group 1 (MB < 50%) (n: 91)	Group 2 (51% < MB < 70%) (n: 41)	Group 3 (MB ≥ 71%) (n: 46)	Total (n: 178)
Non-anginal symptoms, n (%)	31 (34.1)	8 (19.5)	17 (37)	56 (31.5)
Stable angina, n (%)	35 (38.5)	26 (63.4)	20 (43.5)	81 (45.5)
Unstable angina, n (%)	17 (18.7)	6 (14.6)	7 (15.2)	30 (16.9)
NSTEMI, n (%)	8 (8.8)	1 (2.4)	2 (4.3)	11 (6.2)

*MB* myocardial bridge, *NSTEMI* non-ST segment elevation myocardial infarction

Finally, all patients were treated medically for MB and none required surgical treatment.

## Discussion

The results of this study showed the incidence of MB to be 10.2% in patients undergoing RACA, which was significantly higher than that detected with FACCA.

MB is a congenital coronary artery anomaly, which is usually detected incidentally in coronary angiography performed for the diagnosis of coronary atherosclerosis. The incidence of MB differs between autopsy studies (15–85%) and angiographic studies (0.5–2.5%) [10]. Furthermore, the incidence of MB varies according to the coronary angiography method. For example, 0.6% incidence of MB in FACCA was reported by Harikishen et al., whereas Kantarci et al. determined MB incidence of 3.5% in CTCA [14, 15]. In another study, Lu et al. found the incidence of MB to be 6% with conventional coronary angiography, and 30% with CTCA in the same population [11]. The main reason for this difference is the variability in the sensitivity of coronary angiography methods in the detection of MB. Other factors that may affect the incidence of MB are the size and ethnicity of the study population. The incidence of MB was found to be around 1% in FACCA studies performed in Turkish populations similar in size to the current study [16, 17]. In the current study, MB incidences of patients who underwent RACA and FACCA with similar baseline characteristics except for their clinical presentation were compared. The current study demonstrated that the incidence of MB able to be detected on RACA was significantly higher than FACCA. As reflected in the study results, in our clinical practice, femoral access is preferred in myocardial infarctions because we usually use a 7-f introducer due to the complexity of the procedure. The luminal narrowing is dynamic in MB and mild systolic compressions may be overlooked during coronary angiography. In the current study, the main reason for the higher MB incidence compared to FACCA studies was thought to be the routine use of nitroglycerin and diltiazem during the procedure. Nitroglycerin and diltiazem reduce coronary artery resistance [18, 19]. As a result of the synergistic effect of nitroglycerin and diltiazem, even low doses can dramatically modify coronary artery resistance. Nitroglycerin augments vessel wall constriction in patients with MB and previously unseen MBs can appear on coronary angiography after the administration of nitroglycerin. In addition, nitroglycerin increases vascular compliance and facilitates diastolic expansion as well as systolic compression, so MB can be easily detected. Provocation with nitroglycerin has been reported to be safe and useful for the detection of invisible MB [19, 20]. Especially mild degree MBs, which may be overlooked during FACCA, can be detected by nitroglycerin exaggeration on RACA. As a matter of fact, in the current study, about half of the MBs were mild degree. In previous RACA studies, Jiang et al. found the incidence of MB to be 9.4%, while Santos et al. found it to be 8.2% [21, 22]. These rates were close to our results and were quite high compared to FACCA studies. Just like our opinion, they stated that the reason for this high incidence might be the nitroglycerin used during the RACA procedure.

The most common coronary artery affected by MB is the LAD mid-segment, as indicated both in the current study and many other studies [23–26]. Although a previous study from Turkey reported almost equal distribution of MBs in the LAD middle and distal segments and no MB present in the proximal segment, the current study results showed much higher involvement of the LAD mid-segment than proximal and distal segments [27]. Compared to previous FACCA studies, not only the MB incidence but also the number of simultaneously affected coronary arteries was higher in the current study. Although some studies [2, 5, 14, 27] have shown that more than one coronary artery was almost never involved simultaneously, the involvement of two or three coronary arteries together was 5.1% and 2.7%, respectively in the current study. The reason for this, just like the incidence difference, can be attributed to the use of nitroglycerin and diltiazem enabling the detection of previously unseen MBs. In a cadaver study, it was shown that almost all major branches of both coronary arteries were affected and 36% of the samples had more than one MB [5].

There has been considerable controversy regarding the functional significance of MB. Some studies have stated that MB may be associated with ischemia, arrhythmia, and sudden cardiac death, while others have claimed that it is benign and may even protect against atherosclerosis [28–31]. It has been shown that severe and proximal MBs are associated with adverse cardiac events [32, 33]. In the current study, 70% of the patients undergoing RACA for different clinical indications had no significant CAD, which could explain these clinical conditions. MBs may be responsible for the clinical condition of these patients. Ural et al.'s study has shown that MBs can cause stable angina, unstable angina, and non-anginal symptoms [33]. As in the current study, Matta et al. have reported that MB can cause myocardial infarction in patients without significant CAD and may be a cause of MINOCA [34]. Most of the MBs in our study were mild, but it should be kept in mind that the severity of MB may be dynamic depending on the hemodynamic status and the drugs used. Therefore, even angiographically mild MB can lead to clinical symptoms over time. In the current study, 70% of patients with a positive SPECT or treadmill exercise test had no significant CAD that could explain this positivity. One reason for this condition might be false-positive test results, but still the most important reason was considered to be MB-related ischemia. It is generally accepted that MB causes coronary atherosclerosis in the LAD segment proximal to the MB and enhances its natural progression through several different mechanisms [3, 9, 10, 32]. Similar to those studies, 35.7% of MB-related vessels had CAD in the current study, of which more than 80% were proximal to the MB, and the majority were mild.

This study had some important limitations, primarily that it was a retrospective and single-center study. Dedicated clinical studies would be required to support the relationship of MB with clinical symptoms in the results of the current study.

## Conclusion

The results of this study demonstrated that the incidence of myocardial bridge in RACA patients was much higher than the rates reported in current and previous FACCA studies. The LAD mid-segment was the most affected coronary artery. Multiple coronary artery involvement was not uncommon and approximately one-third of the patients with myocardial bridge had CAD proximal to the bridge.

## Data Availability

The datasets used and/or analysed during the current study available from the corresponding author on reasonable request.

## References

[1] Zoghi M, Duygu H, Nalbantgil S (2006). Impaired endothelial function in patients with myocardial bridge. Echocardiography.

[2] Li J, Shang Z, Min Y (2008). Angiographic prevalence of myocardial bridging in a defined very large number of Chinese patients with chest pain. Chin Med J.

[3] Angelini P, Trivellato M, Donis J, Leachman RD (1983). Myocardial bridges: a review. Prog Cardiovasc Dis.

[4] Ishii T, Asuwa N, Masuda S, Ishikawa Y (1998). The effects of a myocardial bridge on coronary atherosclerosis and ischaemia. J Pathol.

[5] Bandyopadhyay M, Das P, Baral K, Chakroborty P (2010). Morphological study of myocardial bridge on the coronary arteries. Indian J Thorac Cardiovasc Surg.

[6] Bauters C, Chmait A, Tricot O (2002). Coronary thrombosis and myocardial bridging. Circulation.

[7] Rossi L, Dander B, Nidasio GP (1980). Myocardial bridges and ischemic heart disease. Eur Heart J.

[8] Ishikawa Y, Kawawa Y, Kohda E (2011). Significance of the anatomical properties of a myocardial bridge in coronary heart disease: a review. Circ J J-Stage.

[9] Möhlenkamp S, Hort W, Ge J, Erbel R (2002). Update on myocardial bridging. Circulation.

[10] Bourassa MG, Butanaru A, Lesparance J, Tardiff JC (2003). Symptomatic myocardial bridges: overview of ischemic mechanisms and current diagnostic and treatment strategies. J Am Coll Cardiol.

[11] Lu GM, Zhang LJ, Guo H (2008). Comparison of myocardial bridging by dual-source CT with conventional coronary angiography. Circ J.

[12] Hwang JH, Ko SM, Roh HG (2010). Myocardial bridging of the left anterior descending coronary artery: depiction rate and morphologic features by dual source CT coronary angiography. Korean J Radiol.

[13] Jolly SS, Amlan S, Hamon M (2009). Radial versus femoral access for coronary angiography or intervention and the impact on major bleeding and ischemic events: a systematic review and meta-analysis of randomized trials. Am Heart J.

[14] Harikrishnan S, Sunder KR, Tharakan J (1999). Clinical and angiographic profile and follow-up of myocardial bridges: a study of 21 cases. Indian Heart J.

[15] Kantarci M, Duran C, Durur I (2006). Detection of myocardial bridge with ECG-gated MDCT and multiplanar reconstruction. AJR.

[16] Oylumlu M, Dogan A, Astarcıoglu MA (2014). Angiographic prevalence of myocardial bridging in our department. Kosuyolu Heart J.

[17] Soran O, Pamir G, Erol C (2000). The incidence and significance of myocardial bridge in a prospectively defined population of patients undergoing coronary angiography for chest pain. Tokai J Exp Clin Med.

[18] Joyal M, Cremer FK, Pieper AJ (1985). Systemic, left ventricular and coronary hemodynamic effects of intravenous diltiazem in coronary artery disease. Am J Cardiol.

[19] Fam MW, McGregor M (1968). Effect of nitroglycerin and dipyridamole on regional coronary resistance. Circulation.

[20] Hongo Y, Tada H, Ito K (1999). Augmentation of vessel squeezing at coronary-myocardial bridge by nitroglycerin: study by quantitative coronary angiography and intravascular ultrasound. Am Heart J.

[21] Jiang X, Zhou P, Wen C (2021). Coronary anomalies in 11,267 Southwest Chinese patients determined by angiography. BioMed Res Int.

[22] Santos LM, Araujo EC, Sousa LNL (2007). Multi-arterial myocardial bridge: uncommon clinical and anatomical presentations. Arq Bras Cardiol.

[23] Geiringer E (1951). The mural coronary. Am Heart J.

[24] Polacek P (1961). Relation of myocardial bridges and loops on the coronary arteries to coronary occlusion. Am Heart J.

[25] Noble J, Bourassa MG, Petitclerc R, Dyrda I (1976). Myocardial bridge and milking effect of the left anterior descending coronary artery: normal variant or obstruction?. Am J Cardiol.

[26] Kramer JR, Kitazume H, Proudfit WL, Sones FM (1982). Clinical significance of isolated coronary bridges: benign and frequent condition involving the left anterior descending artery. Am Heart J.

[27] Cay S, Oztürk S, Cihan G (2006). Angiographic prevalence of myocardial bridging. Anadolu Kardiyol Derg.

[28] Dean JW, Mills PG (1994). Abnormal ventricular repolarization in association with myocardial bridging. Br Heart J.

[29] Faruqui AMA, Maloy WC, Feiner JM (1978). Symptomatic myocardial bridging of coronary artery. Am J Cardiol.

[30] Feldman AM, Baughman KL (1986). Myocardial infarction associated with a myocardial bridge. Am Heart J.

[31] Ishii T, Asuwa N, Masuda S (1991). Atherosclerosis suppression in the left anterior descending coronary artery by the presence of a myocardial bridge: an ultrastructural study. Mod Pathol.

[32] Ishikawa Y, Akasaka Y, Suzuki K (2009). Anatomic properties of myocardial bridge predisposing to myocardial infarction. Circulation.

[33] Ural E, Bildirici U, Celikyurt U (2009). Long-term prognosis of non-interventionally followed patients with isolated myocardial bridge and severe systolic compression of the left anterior descending coronary artery. Clin Cardiol.

[34] Matta A, Canitrot R, Nader V (2021). Left anterior descending myocardial bridge: angiographic prevalence and its association to atherosclerosis. Indian Heart J.

